# Assessing a local vaccination initiative: Eight years of “Immuniser Lyon” in France

**DOI:** 10.1371/journal.pgph.0007468

**Published:** 2026-10-16

**Authors:** Juliette Dessemon, Romain Saux, Christelle Elias, Anne-Sophie Ronnaux-Baron, Anne Sophie Malachane, Sophie Pamies-Jeantet, Benoit Soubeyrand, Pascal Roy, Philippe Vanhems

**Affiliations:** 1 PHE3ID, International Center for Research in Infectiology, Inserm U1111, CNRS UMR5308, ENS de Lyon, University Lyon 1, Lyon, France; 2 Claude Bernard University, Lyon 1 University, Lyon, France; 3 Service Hygiène, Epidémiologie, Infectiovigilance et Prévention, Hospices Civils de Lyon, Lyon, France; 4 Regional health monitoring center, Auvergne-Rhône-Alpes Regional Health Agency, Lyon, France; 5 Academy of Pharmacy, Lyon, France; 6 Doctor, Former Director of Urban Ecology, Lyon, France; 7 Blossom Vaccinology, La Chapelle du Mont du Chat, France; 8 Laboratoire de Biométrie et Biologie Evolutive, CNRS UMR5558, Equipe Biostatistiques Santé, Villeurbanne, France; 9 Service de Biostatistique-Bioinformatique, Hospices Civils de Lyon, Lyon, France; PLOS: Public Library of Science, UNITED STATES OF AMERICA

## Abstract

“Immuniser Lyon” (IL) is a French city-level initiative launched in 2015 to promote vaccination through coordinated, locally tailored actions. The objective of this study is to describe and compare temporal trends in vaccine coverage for HPV and influenza in the Rhône area versus the neighboring Auvergne-Rhône-Alpes (ARA) region, and to explore whether observed patterns are consistent with a potential contribution of the IL initiative. A comparative analysis of vaccine coverage was performed for HPV (2010–2022) and influenza (2016–2020) between different areas within the Auvergne-Rhône-Alpes (ARA) region versus the ARA region, using binomial log-linear models. HPV vaccine coverage in Rhône and ARA followed similar patterns, increasing from 2015 to 2022 after a nationwide decline. For influenza, vaccine coverage among adults aged ≥65 was significantly higher in Lyon in 2020 (64.5%) than in the rest of ARA (59.2%, OR=1.25, 95% CI [1.24–1.26], p < 0.001). While HPV trends reflected broader national dynamics, the observed pattern may be consistent with a potential contribution of IL to the accelerated uptake of influenza vaccines in older adults. These findings highlight the potential of sustained local action to complement national immunization strategies.

## Introduction

Despite national vaccination policies, France continues to face suboptimal vaccination coverage in several populations, and remains one of the countries with the lowest levels of public confidence in vaccines [1–3]. “Immuniser Lyon” (IL), launched in 2015 in the city of Lyon in France, represents a pioneering local response at a large city level: a multi-stakeholder initiative embedded in municipal and departmental health strategies [4,5]. Its founding goal was to develop a territorially coordinated response to vaccine hesitancy and undercoverage.

IL’s approach was grounded in community mobilization. The collective brought together civil society, public institutions, industry, health service users, liberal and academic health professionals. Over eight years, it supported a wide range of actions—from professional training to public information, presence at civic events, and collaborations with schools, pharmacists, and media. It adapted dynamically to contextual shifts, including the 2018 extension of mandatory childhood vaccinations and the COVID-19 pandemic, which prompted a shift toward digital campaigns [6].

IL played a central role in mobilizing local actors, promoting and reinforcing national vaccination messages at the local level. This study aims to describe temporal trends in vaccine coverage and to explore whether observed patterns are consistent with a potential contribution of IL in its territory of action.

## Materials and methods

### Ethics statement

This study used aggregated, anonymized data from publicly available databases and administrative health records. No individual-level identifiable data were accessed. Ethical approval was not required according to national regulations on secondary use of anonymized health data. All procedures complied with the Declaration of Helsinki and relevant data protection laws.

### Data collection and statistical analysis

This ecological time-trend comparative study assessed vaccine coverage trends in the Auvergne-Rhône-Alpes (ARA) region by comparing the Rhône department (1.9 million inhabitants), where the Lyon metropolitan area (1.4 million inhabitants; approximately 73% of the department population) represents the main setting of the IL initiative, with non-exposed neighboring departments (approximately 8 million inhabitants) (Fig 1). Temporal trends in vaccine coverage were compared between areas exposed to the IL initiative and neighboring areas not exposed to IL, using statistical modeling of vaccine coverage and its evolution from 2010 to 2022. This approach allowed us to assess whether vaccine coverage evolved differently over time between the Rhône area and the rest of the region.

**Fig 1:**
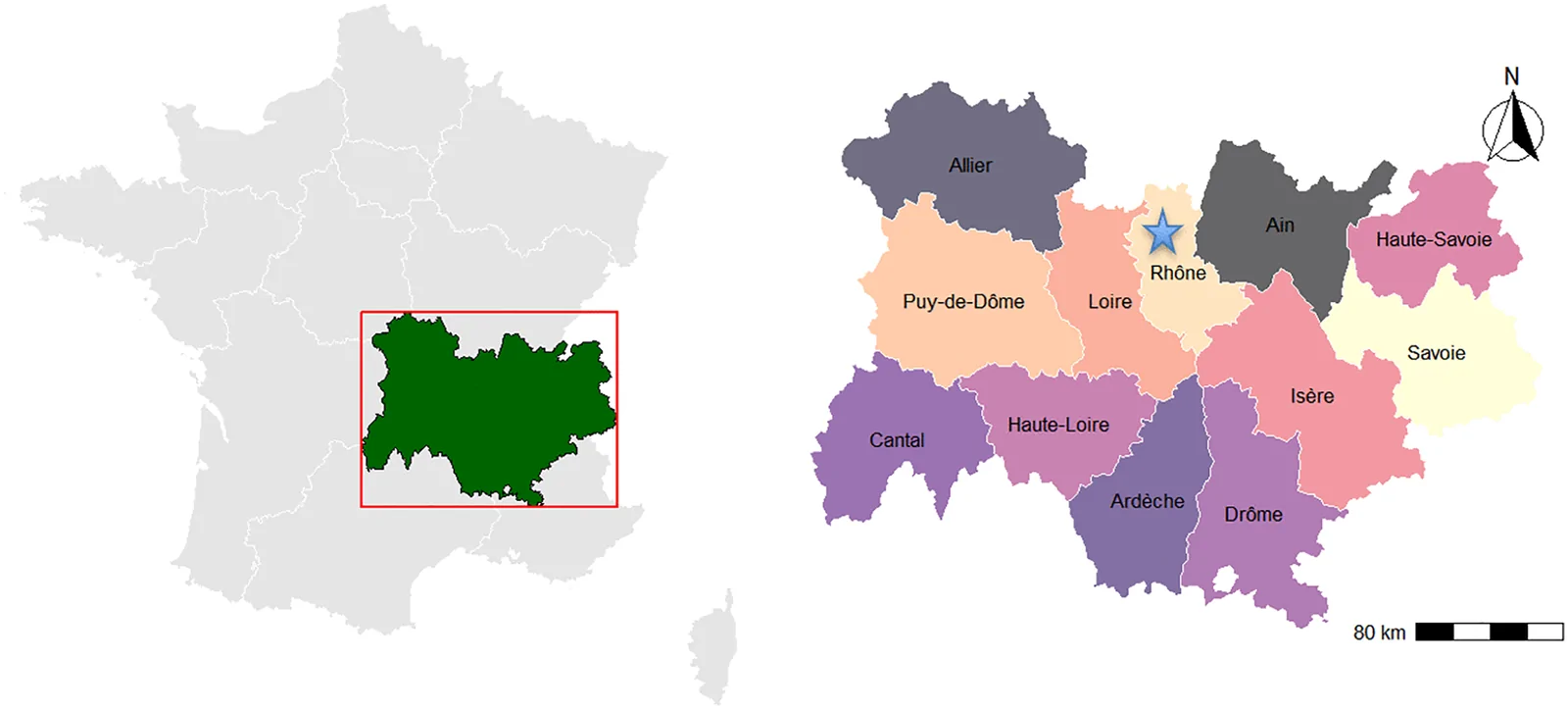
France and the Auvergne-Rhône-Alpes region with departmental division. The “Rhône” area, marked with a star, was the geographic region exclusively exposed to the Immuniser Lyon (IL) initiative. Map created in R using geographic boundary data from france-geojson (G. David; https://github.com/gregoiredavid/france-geojson), derived from IGN Admin Express COG data, under the Open Licence.

The analysis focused on two vaccines—human papillomavirus (HPV) and influenza. These two vaccines were selected because: (1) they target distinct populations with different sociodemographic challenges (adolescent girls for HPV; adults aged ≥65 for influenza); (2) they represent two major vaccination priorities in France during the study period, with known coverage gaps; and (3) their data are available from accessible national surveillance databases.

HPV vaccination coverage was calculated as the proportion of 16-year-old girls who had completed the full vaccination schedule within their birth cohort. In France, the recommended HPV vaccination schedule during the study period consisted of two doses for girls aged 11–14 years (primary schedule) and three doses for girls aged 15–19 years (catch-up schedule). A complete vaccination schedule was defined as receiving all required doses within the cohort birth year. HPV data for cohorts born from 1994 to 2006 were sourced from Santé Publique France (SPF, National Public Health Agency) databases for the period from 2010 to 2022. These data are derived from pharmacist dispensing records extracted from the French National Health Data System (SNDS), specifically its reimbursement database (DCIR), which covers community pharmacy dispensing for virtually the entire French population (~90%). The denominator corresponded to the number of girls in each birth cohort alive at age 16 and was extracted from the National Institute of Statistics and Economic Studies (INSEE) databases for the years 2010–2020; missing data for 2021–2022 were extrapolated by linear regression. Trends and interdepartmental variability were assessed using binomial log-linear models, comparing the Rhône department to the rest of the ARA region, excluding the Isère department due to an independent HPV campaign.

Influenza vaccine coverage was defined as the proportion of individuals aged 65 and over who received at least one dose of seasonal influenza vaccine during the corresponding vaccination campaign (October–March). In France, annual influenza vaccination is recommended for all individuals aged ≥65 years. Data from 2016 to 2020 were obtained from the National Health Data System (SNDS) and Health Information Coordination System (DCIR) databases, with population denominators derived from INSEE; 2020 values were extrapolated linearly. For influenza analyses, vaccination coverage in the Lyon metropolitan area (primary intervention area) was compared with both the broader ARA region and the Rhône outside the metropolis.

Vaccine coverage was analyzed using log-linear effects and cubic B-splines were introduced to assess deviations from log-linearity. The approach was subsequently extended to allow for different temporal evolutions within the initially grouped departments. Additionally, the temporal trend models were compared using the Akaike Information Criterion (AIC), and likelihood ratio tests were used to assess the improvement in fit between nested models (S1 Table). Cross-sectional odds ratios (ORs) comparing vaccine coverage between the intervention area and the comparator were computed for selected years using separate binomial logistic models. The data were then compiled using the statistical software R version 4.3.1.

## Results

In 2010, complete HPV vaccine coverage among 16-year-olds were 24.7% in the Rhône department and 28.3% in the rest of the ARA region, excluding the Isère department (OR= 0.83, 95%CI [0.79;0.88], p < 0.001). Coverage dropped to 13.0% and 12.4% respectively, in 2015 (OR= 1.05, 95%CI [0.98;1.13], p = 0.137), before increasing to 40.5% (Rhône) and 41.5% (rest of the region) in 2022 (OR= 0.96, 95%CI [0.92;1.00], p = 0.065).

Between 2015 and 2022, the annual increase in vaccine coverage corresponded to crude odds ratios (OR) of 1.23 (Rhône) and 1.25 (other departments), equivalent to cumulative crude ORs of 4.21 and 4.64 over the eight years. The application of a model that distinguished the Rhône from the remainder of the ARA region was more flexible, allowing the incorporation of B-spline functions, which yielded a superior fit to that of the previously described model (AIC = 4590.5, Fig 2b). At the same time, exhibiting relatively comparable “V”-shaped trajectories, the vaccine coverage changes across the different departments exhibited substantial heterogeneity (Fig 2c). Consequently, the model was ultimately retained, with distinct B-spline functions fitted for each department (AIC = 1590.8, Fig 2d).

**Fig 2:**
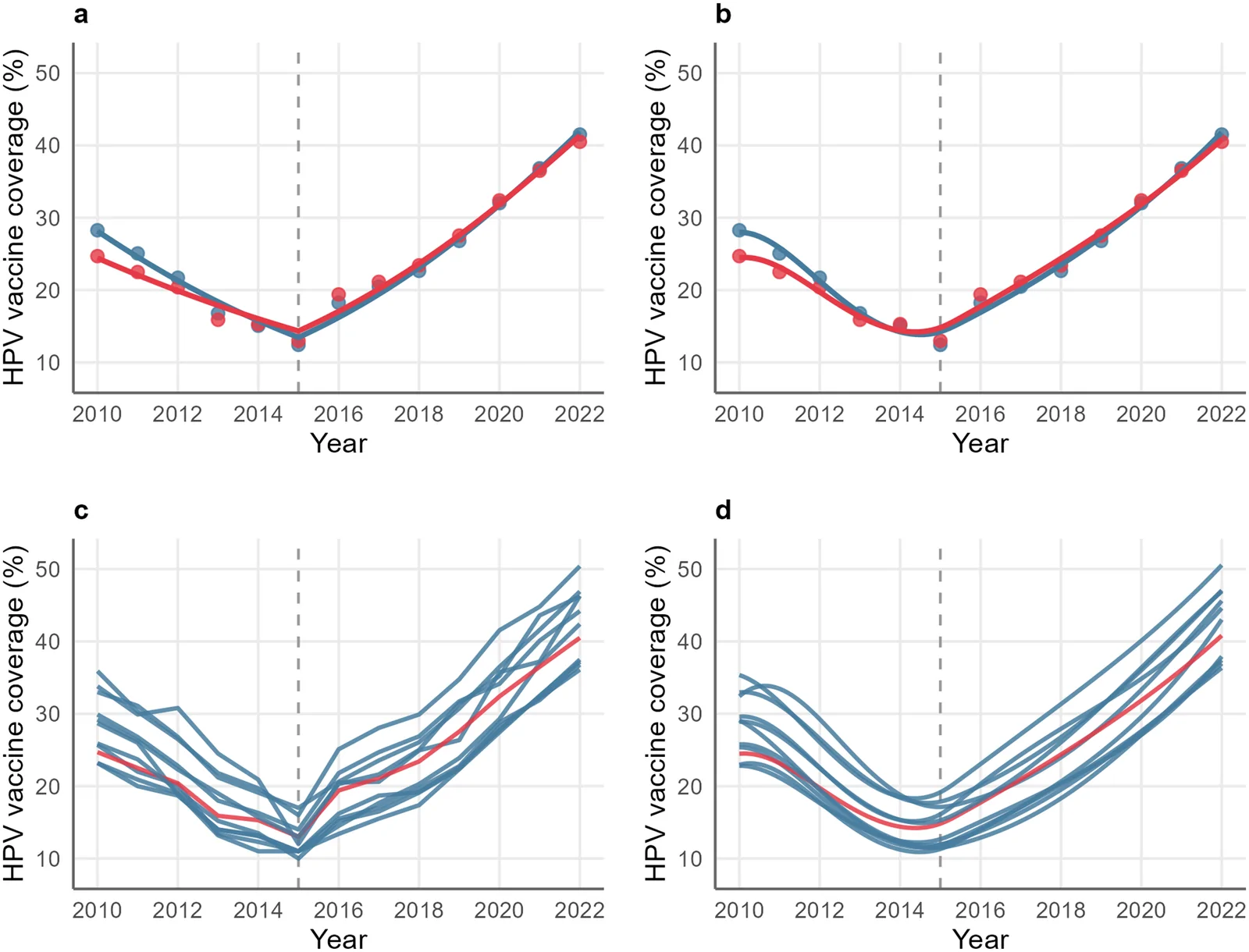
a, b, c, d. Trends in HPV vaccination coverage (%) between the Rhône and the Auvergne-Rhône -Alpes region (excluding Isère), 2010–2022. (a) Log-linear model of complete HPV vaccination coverage in the Rhône department (red) compared to the Auvergne-Rhône-Alpes (ARA) region excluding the Isère department (blue). (b) Same comparison as in panel (a), using B-spline functions to account for non-linear trends. (c) Comparison of the Rhône department (red) with each of the other ARA departments (excluding Isère, blue). (d) Comparison as in panel (c), with separate B-spline functions fitted for each department to capture heterogeneous temporal patterns.

The influenza vaccine coverage for the Lyon metropolitan area (46.7%) did not differ significantly from that of the rest of the ARA Region (46.9%) in 2016 (OR = 0.99, 95% CI [0.98; 1.00], p = 0.142). Conversely, in 2020, the rate was significantly higher (64.5% vs 59.2%) (OR = 1.25, 95% CI [1.24; 1.26], p < 0.001). Fig 3a illustrates the fluctuations and patterns in influenza vaccination coverage among individuals aged 65 and above between 2016 and 2020 for the Lyon metropolitan area and the remainder of the ARA region. The trend model that distinguished the metropolitan area from the rest of the region was more informative than the model that did not differentiate between the two (AIC = 28934 vs. AIC = 34751). The application of B-splines enabled a more flexible modelling of the evolutionary trends, thereby improving the fit (AIC = 27143, Fig 3b). Although the evolutionary trends of vaccine coverage presented relatively similar trajectories, they differed between departments, indicating a strong heterogeneity of evolutionary trends (Fig 3c, AIC = 3082.4). The more flexible modelling of the evolutionary trends of the different departments using B-splines (Fig 3d) provided a superior fit than the previous model (AIC = 991.1).

**Fig 3:**
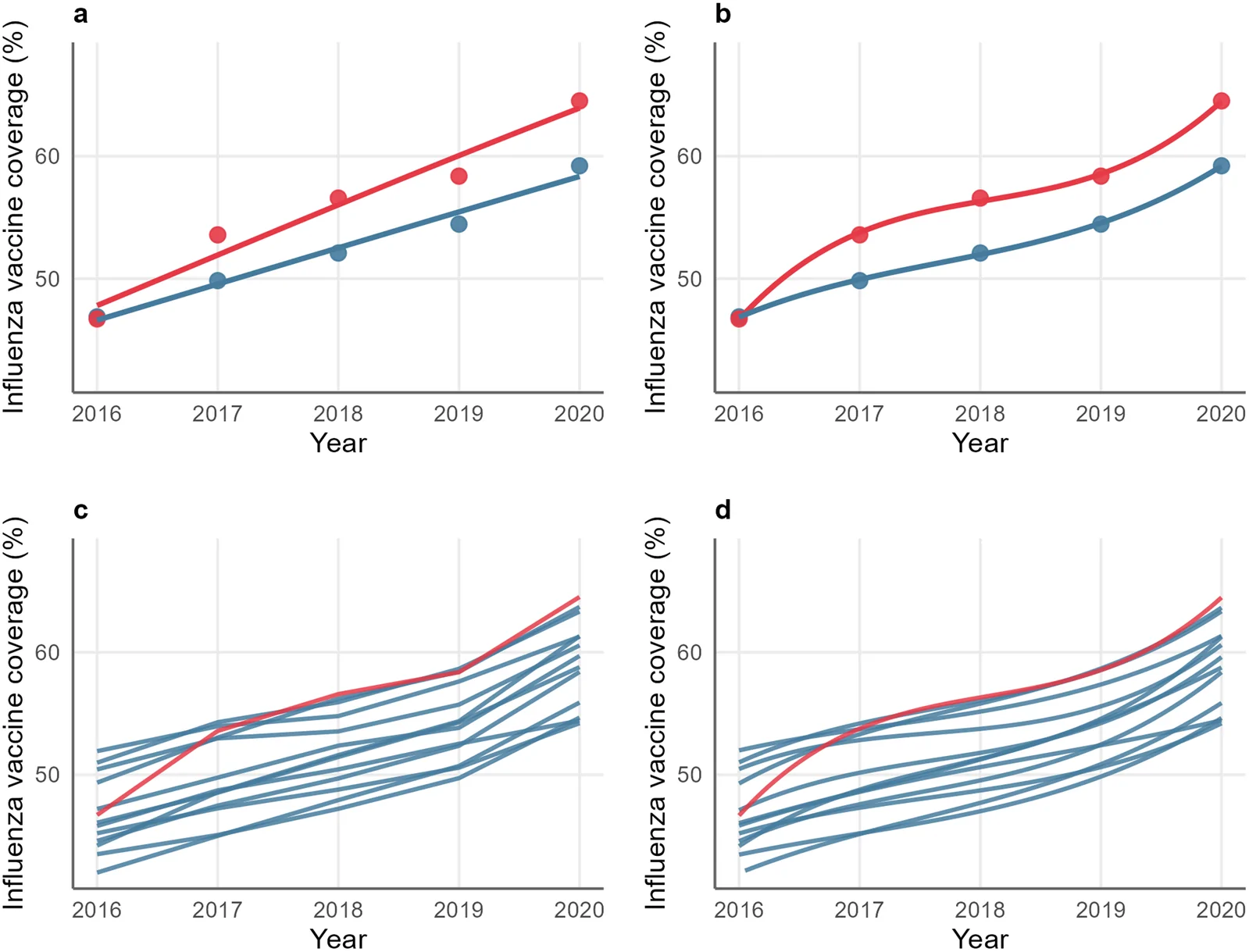
a, b, c, d. Trends in influenza vaccination coverage (%) between the Lyon metropolitan area and the Auvergne-Rhône-Alpes region, 2016–2020. (a) Comparison of influenza vaccination coverage between the Lyon metropolitan area (red) and the Auvergne-Rhône-Alpes region (blue), modeled using a basic log-linear approach. (b) Same comparison as in panel (a), using B-spline functions to capture non-linear trends over time. (c) Comparison of influenza vaccination coverage between the Lyon metropolitan area (red) and each department of the Auvergne-Rhône-Alpes region (blue). (d) Comparison of influenza vaccination coverage between the Lyon metropolitan area (blue) and the departments of the Auvergne-Rhône-Alpes region (black), with B- spline function adjustment.

## Discussion

This evaluation provides one of the first assessments of a city-scale vaccination promotion initiative in France. The temporal trends in HPV and influenza vaccination coverage in the Rhône and the broader ARA region align with national data [7,8].

The comparison of the HPV vaccine coverage in the Rhône with that of the rest of the ARA region did not show any clear divergence in temporal trends. The “V”-shaped curve, which exhibits a trough for young girls born between 1995 and 2002, was observed consistently across the 12 departments and likely reflects the influence of the broader national context. This includes the initial adherence to the 2007 vaccination recommendations, marked by a peak period between 2009 and 2011, during which complete HPV vaccination coverage reached nearly 30% [7]. This was followed by a period of disaffection from 2011, and then renewed adherence, which is likely associated with the publication of reassuring results from tolerance studies [9,10].

Influenza coverage showed no significant differences between Lyon and the rest of the region in 2016, but a marked increase was seen in the Lyon metropolitan area by 2020. These results reflect a trend of increasing influenza vaccine coverage, which has been more significant for the Lyon metropolitan area. While causality cannot be inferred, this pattern may reflect the influence of IL’s targeted efforts—particularly its campaigns for older adults, educators, and public-sector employees, as well as increased use of digital outreach during the COVID-19 pandemic. This period coincided with heightened public awareness of respiratory viral infections during the COVID-19 pandemic, which may have amplified the impact of IL’s digital outreach on influenza vaccination messages among older adults.

This study has several strengths. It relies on robust, standardized indicators— vaccine coverage —which are routinely monitored and closely aligned with the primary objective of the “Immuniser Lyon” initiative. By focusing on two distinct vaccines targeting different populations (adolescents vs. older adults) and involving different sociocultural and organizational challenges, the analysis provides a broader and more nuanced assessment of IL’s potential reach and relevance. In addition, the use of flexible statistical modeling (e.g., B-splines) enabled a detailed analysis of both temporal trends and interdepartmental heterogeneity, going beyond simple geographic comparisons [11]. This analytic depth offers a more granular understanding of how local contexts may shape vaccine coverage patterns, and how IL’s actions may have interacted with those dynamics. Finally, the methodology developed here is reproducible and can serve as a framework for evaluating similar local public health interventions in other areas or countries.

This study has some limitations that must be acknowledged. While relevant and measurable, the choice of vaccination coverage rates as the primary outcome does not capture intermediary outcomes such as vaccine confidence, health literacy, public perception, or the engagement of health professionals—all of which were central to the goals of the IL initiative [12]. As a result, potential effects on knowledge, attitudes, or behavioral intentions remain outside the scope of this analysis.

Similarly, the vaccine coverage data are aggregated and do not allow for individual-level analyses or the assessment of repeated vaccinations (e.g., for influenza), which prevents the interpretation of causality [13]. Differences in spatial scale between analyses (Lyon metropolitan area for influenza vs. Rhône department for HPV, where approximately 27% of the population was not directly exposed to IL) may also limit direct comparability between findings.

Although alternative approaches such as log-binomial or modified Poisson regression with robust variance are commonly recommended to directly estimate risk ratios when outcomes are not rare, these models frequently present convergence difficulties in applied settings, particularly in models with high outcome prevalence or complex functional forms [14,15]. The use of odds ratios from logistic models was therefore retained, acknowledging that they may overestimate the magnitude of associations.

Finally, the observation period—limited to eight years—while sufficient to detect certain trends, may not capture the full impact of a complex and adaptive initiative like IL, whose influence could unfold over a longer horizon. Moreover, this timeframe overlaps with major national events, including the extension of mandatory childhood vaccinations in 2018 and the COVID-19 pandemic beginning in 2020 [16].

Therefore, while our findings suggest a positive association, they should be interpreted with caution, particularly regarding attribution of effects.

This study offers one of the first evaluations of a locally coordinated, multi-stakeholder vaccination initiative in a large French city (1.4 million of inhabitants). While causality cannot be inferred from this ecological approach, the observed patterns suggest that sustained, locally tailored promotion can influence vaccination uptake—particularly when targeting audiences less reached by national campaigns.

This work reflects the inherent challenges and complexities associated with evaluating locally conducted public health actions [17–19]. Although improvements could be made, this preliminary evaluation has enabled the development of a methodology that can be applied to other institutional initiatives. By employing this approach prospectively, it is possible to continue evaluating future actions and extend the evaluation and comparison of similar initiatives to different cities or territories.

## Supporting information

S1 Table Likelihood ratio tests (LRT) for model selection. Nested model comparisons (AIC and p-values) for influenza and HPV vaccination coverage. AIC: Akaike Information Criterion, ARA: Auvergne-Rhône-Alpes.
(DOCX)
