# Supplementary material for: Assessing a local vaccination initiative: Eight years of “Immuniser Lyon” in France
Source: PLOS Glob Public Health. 2026 Oct 16;6(10):e0007468. doi: 10.1371/journal.pgph.0007468 (PMC13674706; doi:10.1371/journal.pgph.0007468)
Supplement: Supplementary file 1 [file pgph.0007468.s001.docx]

**S1 Table**

**Likelihood Ratio Tests (LRT) for model selection**

| Model comparison | p-value | AIC |
| --- | --- | --- |
| Influenza vaccine coverage (2016–2020, individuals aged ≥65 years) | | |
| Null model (common time trend, no territory distinction) | — | 34 751 |
| Null model → Separate log-linear slopes (Lyon metropolitan area vs. rest of ARA) | < 0.001 | 34 751 → 28 934 |
| Separate log-linear slopes → Separate B-spline curves (Lyon metropolitan area vs. rest of ARA) | < 0.001 | 28 934 → 27 143 |
| Separate B-spline curves (2 groups) → Log-linear trend per department | < 0.001 | 27 143 → 3 082 |
| Log-linear trend per department → B-spline curve per department | < 0.001 | 3 082 → 991 |
| HPV vaccine coverage (2010–2022, 16-year-old girls by birth cohort) | | |
| Common log-linear time trend (no territory distinction) | — | 15 240 |
| Common log-linear trend → Common B-spline curve (all departments) | < 0.001 | 15 240 → 4 689 |
| Common B-spline curve → Separate B-spline curves (Rhône vs. remaining departments of ARA) | < 0.001 | 4 689 → 4 591 |
| Separate B-spline curves (2 groups) → B-spline curve per department | < 0.001 | 4 591 → 1 591 |

p-values were computed from the difference in residual deviances between nested models using the chi-squared distribution: p = pchisq(Δdeviance, Δdf, lower.tail = FALSE).

AIC: Akaike Information Criterion, ARA: Auvergne-Rhône-Alpes.
